# Cdon is essential for organ left-right patterning by regulating dorsal forerunner cells clustering and Kupffer’s vesicle morphogenesis

**DOI:** 10.3389/fcell.2024.1429782

**Published:** 2024-08-22

**Authors:** Zhilin Deng, Qin Ran, Wenqi Chang, Chengni Li, Botong Li, Shuying Huang, Jingtong Huang, Ke Zhang, Yuanyuan Li, Xingdong Liu, Yundan Liang, Zhenhua Guo, Sizhou Huang

**Affiliations:** ^1^ Development and Regeneration Key Laboratory of Sichuan Province, Department of Anatomy and Histology and Embryology, School of Basic Medical Sciences, Chengdu Medical College, Chengdu, China; ^2^ Department of Ultrasound, Luzhou People’s Hospital, Luzhou, China; ^3^ Department of Cardiology, Chengdu Seventh People’s Hospital, Chengdu, Sichuan, China; ^4^ Department of Neurology, The Second Affiliated Hospital of Chengdu Medical College, (China National Nuclear Corporation 416 Hospital), Chengdu, China; ^5^ Department of Pathology and Pathophysiology, School of Basic Medical Sciences, Chengdu Medical College, Chengdu, China; ^6^ Ministry of Education Key Laboratory of Child Development and Disorders, China International Science and Technology Cooperation Base of Child Development and Critical Disorders, National Clinical Research Center for Child Health and Disorders, Chongqing Key Laboratory of Pediatrics, Children’s Hospital of Chongqing Medical University, Chongqing, China

**Keywords:** *cdon*, left right patterning, DFCs, KV morphogenesis, cilia, nodal signaling

## Abstract

*Cdon* and *boc* are members of the cell adhesion molecule subfamily III Ig/fibronectin. Although they have been reported to be involved in muscle and neural development at late developmental stage, their early roles in embryonic development remain unknown. Here, we discovered that in zebrafish, *cdon*, but not *boc*, is expressed in dorsal forerunner cells (DFCs) and the epithelium of Kupffer’s vesicle (KV), suggesting a potential role for *cdon* in organ left-right (LR) patterning. Further data showed that liver and heart LR patterning were disrupted in *cdon* morphants and *cdon* mutants. Mechanistically, we found that loss of *cdon* function led to defect in DFCs clustering, reduced KV lumen, and defective cilia, resulting in randomized Nodal/spaw signaling and subsequent organ LR patterning defects. Additionally, predominant distribution of a *cdon* morpholino (MO) in DFCs caused defects in DFC clustering, KV morphogenesis, cilia number/length, Nodal/spaw signaling, and organ LR asymmetry, similar to those observed in *cdon* morphants and *cdon* ^−/−^ embryos, indicating a cell-autonomous role for *cdon* in regulating KV formation during LR patterning. In conclusion, our data demonstrate that during gastrulation and early somitogenesis, *cdon* is essential for proper DFC clustering, KV formation, and normal cilia, thereby playing a critical role in establishing organ LR asymmetry.

## 1 Introduction

The establishment of left-right (LR) patterning is a crucial event in early embryonic development. Organ asymmetry defects are closely related to the occurrence of various congenital diseases such as congenital heart disease and schizophrenia (Huang et al., 2014; Gabriel and Lo, 2020). Although the mechanisms for establishing organ LR patterning are complex and diverse across different animal models (Hamada and Tam, 2020; Onuma et al., 2020; Zhu et al., 2020), the role of the Kupffer’s vesicle (KV)/*Node*-Cilia axis in zebrafish and mice is highly conserved (Grimes and Burdine, 2017; Little and Norris, 2021; Forrest et al., 2022). In zebrafish, the normal development of KV precursor cells, called dorsal forerunner cells (DFCs), and KV morphogenesis/ciliogenesis play crucial roles in initiating left-sided signals in the embryonic lateral plate mesoderm (LPM) (Grimes and Burdine, 2017). During organ LR patterning, DFCs proliferate rapidly during gastrulation movement (Gokey et al., 2015; Abdel-Razek et al., 2023). After the bud stage, DFCs differentiate to form the KV and produce cilia, which oscillate to generate a counter-clockwise liquid flow and initiate specific asymmetric *Nodal/spaw* expression on the LPM (Bakkers et al., 2009; Liu et al., 2019). Furthermore, the left-sided *Nodal/spaw* and its downstream genes *pitx2* and *lft2* are amplified in the LPM. The embryonic midline (such as *shh* and *ntl*) also impedes the expansion of left-sided *Nodal* signals to the right side of the embryo, thus stabilizing the left-sided asymmetric *Nodal* signals (Burdine and Grimes, 2016). Finally, at the late stage of organ development, the left-sided *Nodal* directs an asymmetric migration of organ precursors to establish organ asymmetry patterning (Kajikawa et al., 2022).

The role of zebrafish KV morphogenesis/ciliogenesis and the underlying molecular mechanisms during organ LR patterning have received extensive attention. It has been found that many classical signaling pathways, such as Wnt and Fgf (Neugebauer et al., 2009; Xu et al., 2010; Caron et al., 2012; Zhang et al., 2012) and other genes, such as *Rab5* (Kuhns et al., 2019) and *FOR20* (Xie et al., 2019), participated in KV morphogenesis or ciliogenesis. Our previous studies found that during zebrafish gastrulation, inhibition of retinoic acid (RA) increases the expression of *fgf8* in DFCs, leading to longer cilia and cilia motility disorders (Huang et al., 2011). More recently, we found that the chemokine receptor *cxcr4a* is highly expressed in DFCs, and that *cxcr4a* promotes CyclinD1 expression to regulate ciliogenesis by regulating the phosphorylation of ERK1/2 (Liu et al., 2019). Although many genes have been reported to play crucial roles during KV morphogenesis and ciliogenesis, the mechanisms underlying these processes are far from being elucidated.

**FIGURE 1:**
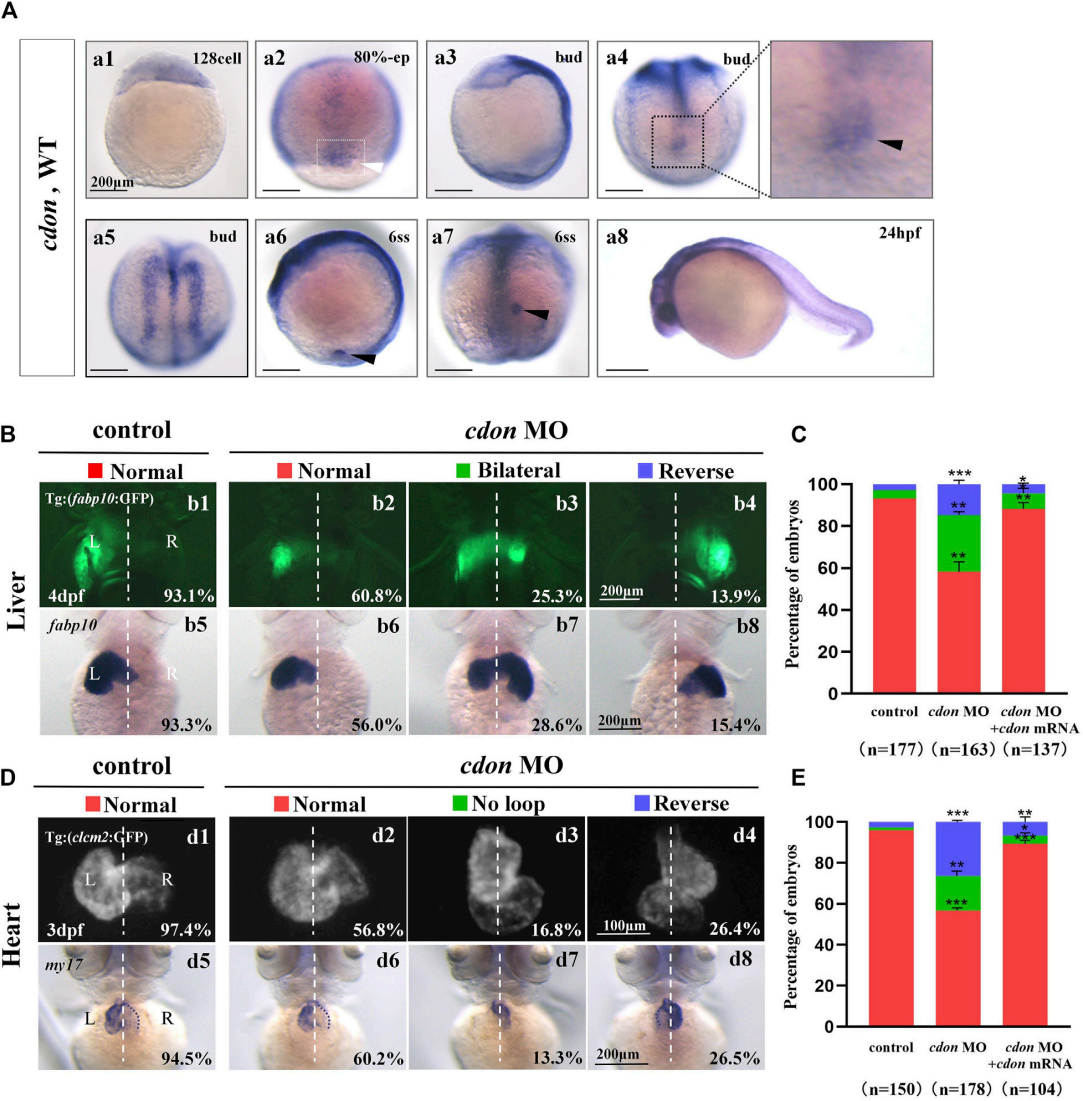
Organ left-right patterning defects in embryos injected with *cdon* MO at the 4-cell stage **(A)** The expression of *cdon* was examined using whole mount *in situ* hybridization from 128-cell stage to 24hpf. *Cdon* was expressed at 128-cell stage (Aa1). White arrowhead showed the expression of *cdon* in DFCs at 80% epiboly stage (Aa2). Black arrowhead showed the expression of *cdon* in DFCs at bud stage (Aa3-a4). *Cdon* was also expressed in midline and presumptive neural crest (Aa5). Black arrow head showed the expression of *cdon* in epithelial cell in KV at 6ss (Aa6-a7). Expression of *cdon* was examined at 24 hpf (Aa8). **(B)** Embryos injected with *cdon* MO displayed liver LR defects. b1, normal liver in *Tg (fabp10:GFP)* transgenic controls (93.1%, n = 102); b2, normal liver in embryos injected with *cdon* MO (60.8%, n = 79); b3, liver bifida in embryos injected with *cdon* MO (25.3%, n = 79); b4, reversed liver in embryos injected with *cdon* MO (13.9%, n = 79). b5, normal liver in wild type controls (93.3%, n = 75); b6-b8, embryos injected with *cdon* MO were examined at 4dpf using *in situ* experiments (n = 84). **(C)** Percentages of normal liver, liver bifida, and reversed liver in embryos used as control, embryos injected with *cdon* MO and embryos co-injected with *cdon* MO and *cdon* mRNA, in respectively. Embryos injected with *cdon* MO show a statistically significant difference compared to controls, and embryos co-injected with *cdon* MO and *cdon* mRNA show a statistically significant difference compared to those injected with *cdon* MO alone. **(D)** Embryos injected with *cdon* MO displayed heart LR defects. d1, normal-loop in *Tg(cmlc2:GFP)* transgenic controls (97.4%, n = 77); d2, normal-loop in embryos injected with *cdon* MO (56.8%, n = 95); d3, no loop in embryos injected with *cdon* MO (16.8%, n = 95); d4, reversed-loop in embryos injected with *cdon* MO (26.4%, n = 95); d5, normal-loop in wild type controls (94.5%, n = 73); d6-d8, wild-type embryos injected with *cdon* MO at the 4-cell stage were examined for cardiac looping at 72 hpf by WISH against *my17* (n = 83). The blue dashed line indicates the atrial edge. **(E)** Percentages of normal looping, no looping, and reversed looping of the heart in embryos being as control, embryos injected with *cdon* MO, and embryos co-injcted with *cdon* MO and *cdon* mRNA. *Cdon* mRNA injection partially rescued the heart LR defect in embryos injected with *cdon* MO (E, the right column showing). Embryos injected with *cdon* MO show a statistically significant difference compared to controls, and embryos co-injected with *cdon* MO and *cdon* mRNA show a statistically significant difference compared to those injected with *cdon* MO alone. Notice: in “C” and “E,” all the transgenic embryos and wild type embryos were used together to calculate the percentage. Statistical analysis was performed using Student’s t-test. “*”*p* < 0.05, “**” *p* < 0.01, “***” *p* < 0.001. Notice: “control” refers to wild-type embryos that were not injected with *cdon* MO.

Cell-adhesion molecule-related/downregulated by oncogenes (*cdon*) and its paralog *boc* [Brother of *cdon* (Kang et al., 2002)] are two members of the cell adhesion molecule subfamily III Ig/fibronectin (Sanchez-Arrones et al., 2012; Jeong et al., 2014). They have been reported to synergistically or individually regulate the development of different organs or diseases (Zhang et al., 2011; Sanchez-Arrones et al., 2012; Jeong et al., 2014; Jeong et al., 2017; Lencer et al., 2023). Regarding *cdon*, early reports showed that mouse *cdon* regulates skeletal muscle and central nervous system development via Shh and N-cadherin/cell adhesion (Cole et al., 2004; Zhang et al., 2006a; Zhang et al., 2006b; Jeong et al., 2014). More detailed studies have shown that *cdon* binds to N-cadherin and induces p38/MAPK signaling to guide cell differentiation and apoptosis during muscle formation and neural differentiation (Lu and Krauss, 2010). In mouse, *Cdon* has been reported to negatively regulate the Wnt signaling pathway during forebrain development, promoting neural differentiation and ventral cell fate determination via interaction with LRP6 (Jeong et al., 2014). Further, researchers found that the localization of Cx43 protein in cells was disorganized in *cdon* mutants, resulting in cardiac structural and functional lesions (Jeong et al., 2017). Regarding *boc*, early reports showed that it plays a role in axon guidance and neuron growth (Connor et al., 2005; Okada et al., 2006). Recently, Lencer et al. found that in zebrafish, *cdon* and *boc* double mutants display trunk neural crest cell (tNCC) migratory defects and loss of slow-twitch muscle, suggesting a non-cell-autonomous role for *cdon* and *boc* in regulating tNCC migration (Lencer et al., 2023). Although *cdon* and *boc* have been reported to play important roles in various contexts in mouse and zebrafish, their roles in early embryonic development remain unknown. Our data showed that during early embryonic development in zebrafish, *cdon*, but not *boc*, is expressed in DFCs during gastrulation movement and in the epithelial cells of KV at the early somitogenesis stage (Figure 1; Supplementry Figures S1). Additionally, *cdon* has been reported to control cell apoptosis and migration in various cell types (Sanchez-Arrones et al., 2012; Chapouly et al., 2020; Lencer et al., 2023). These findings suggest that *cdon* may be involved in regulating the clustering movement of DFCs and the subsequent organ LR patterning in zebrafish. Here, our data identify that *cdon* regulates organ LR patterning via the KV/Cilia-Nodal/spaw cascade during early embryonic development.

## 2 Methods and materials

### 2.1 Zebrafish

Zebrafish wild type (AB), *Tg* (*cmcl2*:*GFP*) (Liu et al., 2022), *Tg* (*fabp10*:*GFP*) (Liu et al., 2022) and *Tg* (*sox17*:*EGFP*) (Chung and Stainier, 2008) lines were maintained at 28.5°C. The embryonic stages were determined according to external morphology, based on the standard criteria described previously (Kimmel et al., 1995).

### 2.2 Whole-mount *in situ* hybridization (WISH)

Embryos used for whole-mount *in situ* hybridization (WISH) were collected at desired stages and fixed in 4% PFA at 4°C overnight. The embryos were then washed with PBST (2 times, 5 min each), dehydrated with 100% MeOH, and stored in MeOH at −20°C. The WISH procedure followed the experimental protocol described previously (Huang et al., 2011; Zhu et al., 2019). The antisense probes *my17*, *fabp10*, *spaw*, *lefty1*, *lefty2*, *sox17*, and *sox32* were prepared as previously described (Zhu et al., 2019). To prepare the *cdon* antisense probe, the cDNA of *cdon* was amplified using PCR and then cloned into the pCS2 + vector. The detailed process can be found in the section “Plasmid Construction.” This construct was then used as the template to synthesize the *cdon* antisense probe according to the previously established protocol (Zhu et al., 2019).

### 2.3 Immunostaining

The immunostaining was performed as previously reported (Zhu et al., 2019). Briefly, the embryos were fixed with 4% PFA at 4°C overnight, then dehydrated with a methanol gradient and stored at −20°C. After methanol/PBST gradient rehydration, PBTN (4% BSA, 0.02% NaN3, PT) was added, and the embryos were incubated at 4°C for 3 h. Then, the α-tubulin antibody (Sigma, T7451, diluted with PBTN at 1:100) was added and incubated overnight on a shaker at 4°C. The next day, the embryos were washed with PT (0.3% Triton X-100 in 1X PBS) 4 times (30 min each), and the secondary antibody, Goat anti-Mouse IgG (H + L) Alexa Fluor 594 (Sigma, SAB4600105), was added (diluted with PBTN at 1:1,000) for overnight incubation in darkness. Finally, the embryos were rinsed with PT more than 6 times (30 min each), and imaging was performed.

### 2.4 Plasmids construction

Total RNA was extracted from zebrafish embryos at 24 hpf according to the reagent instructions (TRIzol, Ambion No.15596-026). cDNA was prepared using the RevertAid First Strand cDNA Synthesis Kit (Fermentas No. K1622) according to the manufacturer’s instructions. The cDNA of *cdon* was amplified using PCR (PrimeSTAR Max Premix, Takara No. R045 A) and cloned into the vector pCS2+ to generate the expression constructs (5x In-Fusion HD Enzyme Premix, Takara No. 639649). The primers for cloning were as follows:

PCS^2+^_F: 5′-CTC​GAG​CCT​CTA​GAA​CTA​TAG​TG-3′

PCS^2+^_R: 5′-TGG​TGT​TTT​CAA​AGC​AAC​GAT​ATC​G-3′

Cdon_F: 5′-TCT​TTT​TGC​AGG​ATC​CGT​GAA​ACA​GCG​TCA​TGG​AGG​AC-3′

Cdon_R: 5′-GTT​CTA​GAG​GCT​CGA​GTG​TTC​AGA​TCT​CCT​GCA​CGG​T-3′

### 2.5 MO and mRNA injection

The antisense *cdon* ATG morpholino oligonucleotide (*cdon* MO^atg^) was synthesized (Gene Tools) and applied to block *cdon* mRNA translation. *cdon* MO^atg^: 5′-ATA​ATC​TCA​GGC​CAC​CGT​CCT​CCA​T-3′, 400 μM (Cardozo et al., 2014). *Cdon* MO was injected into the yolk of zebrafish embryos at the 1–4 cell stage to block the translation of *cdon* in whole embryos, or at the 256–512 cell stage to specifically block the translation of *cdon* mRNA in DFCs. The *cdon* mRNA was synthesized *in vitro* using the mMESSAGE Kit (AM1340, Ambion). In rescue experiments, the concentration for *cdon* mRNA injection was 15 ng/μL.

### 2.6 Generation of *cdon* ^*−/−*^ mutants

To generate *cdon* ^−/−^ mutants, we used the CRISPR design tool (http://crispr.mit.edu) to design CRISPR short guide RNAs (sgRNAs) targeting exon 3 of the *cdon* gene. Injection mixtures contained 500 pg Cas9 mRNA and 120 pg gRNA in each embryo. Embryos injected with sgRNA and Cas9 mRNA generated the F0 line with mosaic mutations. These F0 mosaic adult fish were outcrossed with wild-type fish to produce F1 heterozygous lines. In one F1 adult, the sequence “AAGGGC” in exon 3 of the *cdon* gene was changed to “TTGATGAATGGGG”, resulting in a truncated Cdon protein. This F1 heterozygote was outcrossed with wild-type fish to produce F2 heterozygous fish, then incrossed them to obtain F3 homozygous mutants. The homozygous embryos from this *cdon* ^*−/−*^ mutant line were used to perform experiments as required.

### 2.7 Imaging

Images of the whole-mount *in situ* hybridization (in 100% glycerol) were taken with an OLYMPUS SZX16 microscope at room temperature. Live embryos of the transgenic line *Tg* (*sox17*:*EGFP*) were placed in 1% low-point melting agarose (LPM agarose) and DFCs were photographed using a confocal microscope (OLYMPUS Fluview FV1000). To obtain images of cilia for immunostained embryos, the embryos were oriented correctly in 1% LPM agarose, and the cilia were photographed using a confocal microscope (OLYMPUS Fluview FV1000).

### 2.8 Statistical analysis

The data were statistically analyzed using GraphPad Prism 9 and ImageJ software. The length of the cilia was measured using a confocal microscope (OLYMPUS Fluview FV1000), and the OLYMPUS SZX16 microscope was used to measure the KV lumen area. The statistical results are presented as the mean ± SEM of three independent experiments. Statistical comparisons between two groups were performed using Student’s t-test. Statistical significance was defined as *p < 0.05, **p < 0.01, ***p < 0.001, and ****p < 0.0001.

### 2.9 Ethics statement

The study was approved by the Institutional Review Board of Chengdu Medical College (SYXK(川)2015–196), and zebrafish were maintained in accordance with the Guidelines of Experimental Animal Welfare from the Ministry of Science and Technology of the People’s Republic of China (2006).

## 3 Results

### 3.1 Heart and liver LR patterning was disturbed in *cdon* morphants

In zebrafish, *cdon* and *boc* have been reported to be involved in trunk neural crest cell (NC) migration and correct proximo-distal patterning in eye development (Cardozo et al., 2014; Lencer et al., 2023), demonstrating the crucial role of *cdon* in zebrafish organ development. Since *cdon* and *boc* synergistically regulate NCs migration (Lencer et al., 2023), facial and eye development (Zhang et al., 2011), to study the role of *cdon* in more early embryonic development, we examined the detailed expression pattern of *cdon* and *boc* from 128-cell stage to 24 hpf. The data showed that *cdon* is a maternal factor that is distributed unequally in cells (Figure 1Aa1). During gastrulation, besides its expression in the presumptive neural crest and midline [Figure 1Aa5, (Jeong et al., 2014)], *cdon* was also highly expressed in DFCs (Figure 1Aa2–a4). At the 6-somite stage, *cdon* was enriched in the epithelium of the KV (Figures 1Aa6, a7). In contrast, although *boc* was maternally and ubiquitously expressed during gastrulation (Supplementry Figures S1A, B), it was not enriched in DFCs or the epithelium of the KV (Supplementry Figures S1E, F). Since disrupting DFC development and the sequential KV/ciliogenesis leads to organ LR patterning defects (Xie et al., 2019; Zhu et al., 2019), we proposed that *cdon* is involved in organ LR patterning. To preliminarily and rapidly evaluate this hypothesis, an ATG MO for *cdon* was used to downregulate the function of *cdon* (Jeong et al., 2014), and organ LR patterning was examined. The results showed that in *Tg (fabp10:GFP)* transgenic embryos and wild type embryos, injection of *cdon* MO did not lead to embryonic defects (Supplementary Figure S2), but gave rise to liver LR patterning defects (Figures 1Bb1–b8, C). Next, we examined whether injection of *cdon* MO led to heart looping defects. The results showed that injection of *cdon* MO caused heart LR patterning defects (Figures 1D, E); many *cdon* morphants displayed linear heart (Figures 1Dd3, d7, E) and reversed heart looping (Figures 1Dd4, d8, E). To further confirm the role of *cdon* in liver and heart LR patterning, we evaluated whether injection of *cdon* mRNA could rescue the organ LR patterning defects in *cdon* morphants. The data showed that injection of *cdon* mRNA partially restored organ LR patterning defects in embryos injected with *cdon* MO (Supplementary Figure S3; Figures 1C, E; the right columns show). These data indicate that *cdon* plays a critical role in organ LR patterning in zebrafish.

### 3.2 Left-sided *Nodal/spaw* cascade is randomized in *cdon* morphants

**FIGURE 2:**
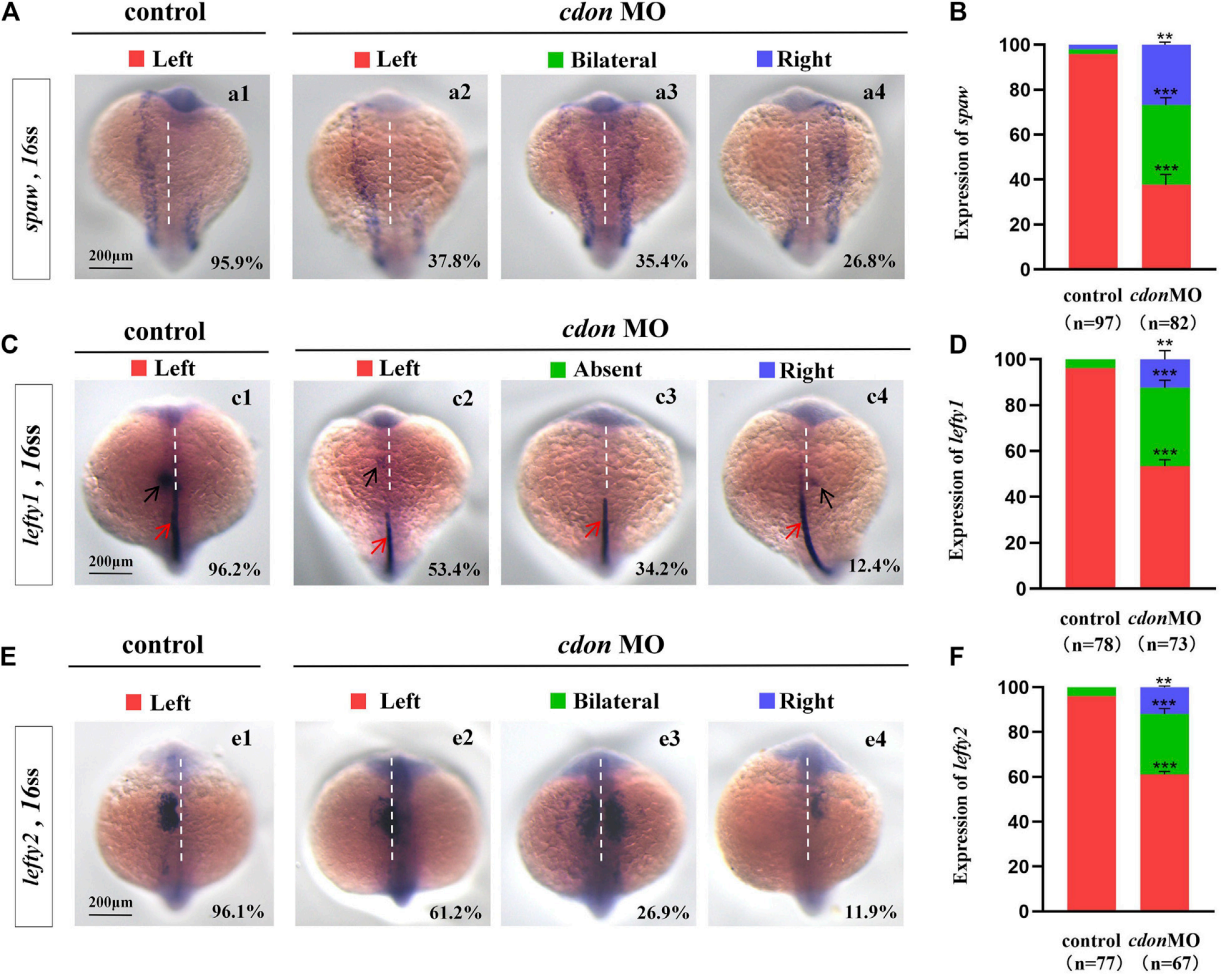
Expression of left-sided Nodal signaling in controls and embryos injected with *cdon* MO **(A,B)** Expression of *spaw* was evaluated in different groups of embryos. a1, left-sided *spaw* in controls (95.9%, n = 97); a2, left-sided *spaw* in embryos injected with *cdon* MO (37.8%, n = 82, *p* < 0.001); a3, bilateral *spaw* in embryos injected with *cdon* MO (35.4%, n = 82, *p* < 0.001); a4, right-sided *spaw* in embryos injected with *cdon* MO (26.8%, n = 82, *p* < 0.01). **(C,D)** *Lefty1* is expressed in the heart field (black arrow) and trunk midline (red arrow) in controls and embryos injected with *cdon* MO. c1, left-sided *lefty1* in embryos being as controls (96.2%, n = 78, black arrow showed); c2, left-sided *lefty1* in embryos injected with *cdon* MO (53.4%, n = 73, *p* < 0.001, black arrow showed); c3, absent *lefty1* in embryos injected with *cdon* MO (34.2%, n = 73, *p* < 0.001); c4, right-sided *lefty1* in embryos injected with *cdon* MO (12.4%, n = 73, *p* < 0.01, black arrow showed) **(E,F)** *Lefty2* is examined in control embryos and embryos injected with *cdon* MO. e1, left-sided *lefty2* in controls (96.1%, n = 77); e2, left-sided *lefty2* in embryos injected with *cdon* MO (61.2%, n = 67, *p* < 0.001); e3, bilateral expression of *lefty2* in embryos injected with *cdon* MO (26.9%, n = 67, *p* < 0.001); e4, right-sided *lefty2* in embryos injected with *cdon* MO (11.9%, n = 67, *p* < 0.01). Statistical analysis was performed using Student’s t-test. “**” *p* < 0.01, “***” *p* < 0.001. Notice: “control” refers to wild-type embryos that were not injected with *cdon* MO.

The crucial role of *Nodal/spaw* in LR patterning has been demonstrated in previous studies (Raya and Izpisua Belmonte, 2006; Speder et al., 2007; Kang et al., 2013). Disruption of *Nodal/spaw* leads to organ laterality defects (Huang et al., 2011). However, in some types of mutants with organ LR patterning defects, *Nodal/spaw* was not disturbed (Trinh and Stainier, 2004; Kurpios et al., 2008; Yin et al., 2010; Huang et al., 2014). To reveal how *cdon* regulates organ laterality, we examined whether left-sided *Nodal/spaw* was disturbed in embryos injected with *cdon* MO. The data showed that left-sided *Nodal/spaw* was disturbed in *cdon* morphants, displaying both-sided and right-sided(Figures 2Aa2–a4, B). Furthermore, we sequentially examined *lefty1* and *lefty2*, the downstream genes of *spaw* in *cdon* morphants. The data showed that the left-sided expression patterns of *lefty2* and *lefty1* in the heart field were also perturbed (Figures 2Cc2–c4, D, Ee2–e4, F). Since *cdon* was also expressed in the midline [Figure 1Aa5, (Jeong et al., 2014)], we observed whether *lefty1* expression in the midline was affected. The data showed that *lefty1* expression in the midline was not affected (Figures 2Cc2–c4, red arrow). These data suggest that the left-sided *Nodal/spaw* cascade was disturbed in *cdon* morphants, which may lead to organ LR patterning defects in *cdon* morphants.

### 3.3 DFCs clustering, KV morphogenesis and cilia are disturbed in *cdon* morphants

**FIGURE 3:**
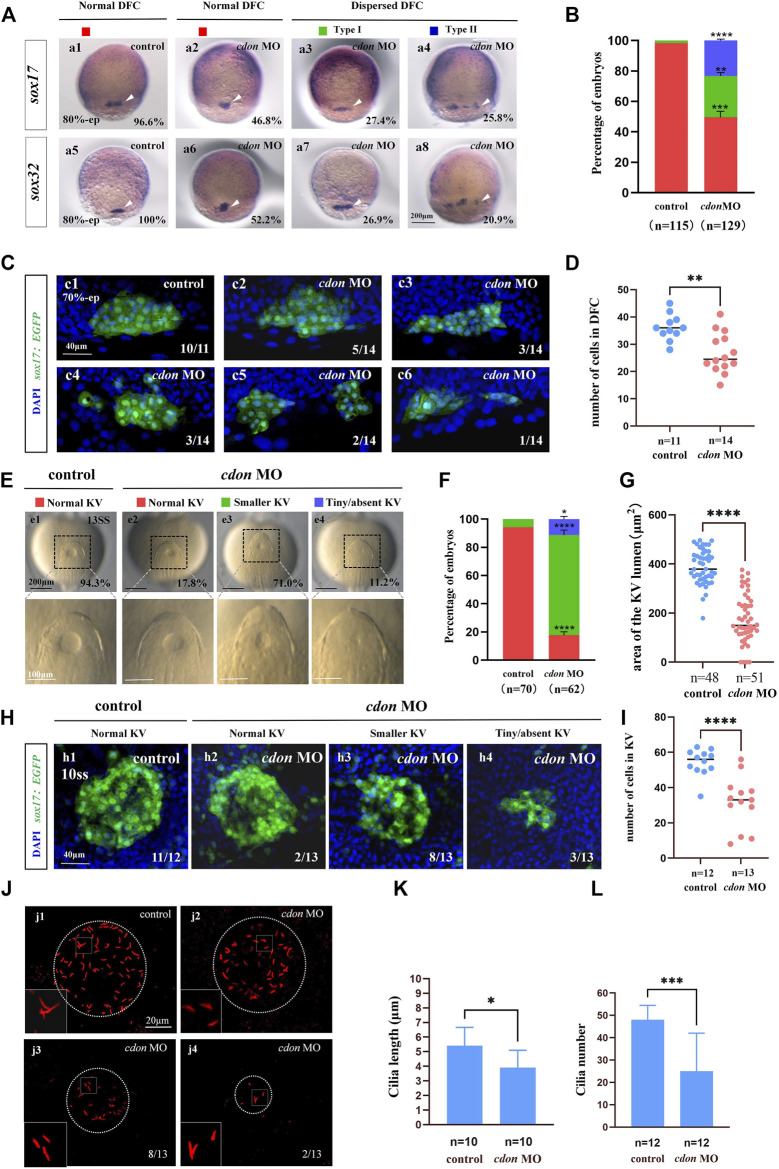
Cdon loss of function disturbs clustering DFCs, KV morphogenesis and cilia **(A)** Expression of *sox17* and *sox32* was examined using WISH at 80% epiboly (white arrow). a1, normal expression of *sox17* in controls (96.6%, n = 58); a2, normal expression of *sox17* in embryos injected with *cdon* MO at the 4-cell stage (46.8%, n = 62); a3, dispersed expression of *sox17* (type I) in embryos injected with *cdon* MO at the 4-cell stage (27.4%, n = 62); a4, dispersed expression of *sox17* (type II) in embryos injected with *cdon* MO at the 4-cell stage (25.8%, n = 62). a5, normal expression of *sox32* in controls (100%, n = 57); a6, normal expression of *sox32* in embryos injected with *cdon* MO at the 4-cell stage (52.2%, n = 67); a7, dispersed expression of *sox32* (type I) in embryos injected with *cdon* MO at the 4-cell stage (26.9%, n = 67); a8, dispersed expression of *sox32* (type II) in embryos injected with *cdon* MO at the 4-cell stage (20.9%, n = 67). **(B)** Statistical analysis was performed for the expression of *sox17* and *sox32* in controls and embryos injected with *cdon* MO. Here all the embryos staining with *sox17* or *sox32* were used together to calculate the percentage. Normal DFC and dispersed DFC show significant differences between control embryos and embryos injected with *cdon* MO at the 4-cell stage. **(C)** Using *Tg (sox17:EGFP)* transgenic zebrafish to detect DFC cell migration at the 70% epiboly stage in both control embryos and embryos injected with *cdon* MO at the 4-cell stage. c1, the DFCs in control embryos are tightly clustered (10/11, n = 11); c2, tightly clustered DFCs in embryos injected with *cdon* MO at the 4-cell stage (5/14, n = 14). c3, in embryos injected with *cdon* MO at the 4-cell stage, the DFCs are arranged in an elongated pattern with a reduced number of cells (3/14, n = 14). c4, mildly dispersed DFCs in embryos injected with *cdon* MO at the 4-cell stage (3/14, n = 14). c5, moderately dispersed DFCs in embryos injected with *cdon* MO at the 4-cell stage (2/14, n = 14). c6, in embryos injected with *cdon* MO at the 4-cell stage, the DFCs are dispersed, and the number of cells is significantly reduced (1/14, n = 14). **(D)** Number of cells in DFC in control and *cdon* MO-injected embryos. “n” represents the sample size. **(E,F)** KV morphology was evaluated. e1, normal KV in embryos in controls (94.3%, n = 70); e2, normal KV in embryos injected with *cdon* MO at the 4-cell stage (17.8%, n = 62, *p* < 0.001); e3, smaller KV in embryos injected with *cdon* MO at the 4-cell stage (71.0%, n = 62, *p* < 0.0001); e4, tiny/absent KV in embryos injected with *cdon* MO at the 4-cell stage (11.2%, n = 62, *p* < 0.05). “Normal KV” represents a KV lumen area greater than 300 μm^2^, “smaller KV” represents a KV lumen area between 100 and 300 μm^2^, and “tiny/absent KV” represents a KV lumen area less than 100 μm^2^. **(G)** Area of the KV lumen (µm^2^) in control and *cdon* MO-injected embryos. “n” represents the sample size. **(H)** The number of epithenium cells in KV were measured on confocal images. **(I)** Number of epithenium cells in KV in control and *cdon* MO-injected embryos. “n” represents the sample size. **(J)** Number and length of cilia were evaluated in controls and embryos injected with *cdon* MO. j1, cilia in embryos being as controls; j2-j4, cilia in embryos injected with *cdon* MO at the 4-cell stage. **(K)** Statistical chart for cilia length in KV. “n”represents the sample size. **(L)** Statistical chart for cilia number in KV. “n”represents the sample size. Statistical analysis was performed using Student’s t-test. “*” *p* < 0.05, “**” *p* < 0.01, “***” *p* < 0.001, “****” *p* < 0.0001. Notice: “control” refers to wild-type embryos that were not injected with *cdon* MO.

Our current research showed that *cdon* was expressed in the DFCs during the gastrulation stage and in the epithelial cells of the KV (Figures 1Aa2–a7). In zebrafish, DFCs form the KV at the early somite stage. Defective KV morphogenesis or ciliogenesis can lead to disturbed left-sided *Nodal/spaw* expression and subsequent organ LR defects (Long et al., 2003; Essner et al., 2005; Wang et al., 2011). To determine whether *cdon* regulates DFC development, KV morphogenesis, or ciliogenesis, we analyzed the expression of DFC markers *sox17* and *sox32*, as well as KV morphogenesis and ciliogenesis in *cdon* morphants. Compared with control embryos, at the 80% epiboly stage, the clustering DFC was disturbed in *cdon* morphants (Figures 3Aa2–a4, Aa6–a8, B), displaying linear expression (Figures 3Aa3, Aa7) and dispersed expression (Figures 3Aa4, Aa8). To further verify whether clustering DFC is affected, we injected *cdon* MO into *Tg(sox17:EGFP)* embryos. The results showed that at the 70% epiboly stage, DFCs remained as a tight cluster in the control embryos, while linear (Figure 3Cc3) or fragmented DFC clusters (Figure 3Cc4–c6) were detected in the *cdon* morphants (Figure 3C). Additionally, there was a significant decrease in the number of DFC cells at the 70% epiboly stage in the embryos injected with *cdon* MO compared to the control embryos (Figure 3D). Further, at the 10–13 somite stage (SS), the size of the KV lumen was smaller in the majority of *cdon* morphants (Figure 3Ee3, F, G), and a small portion of morphants displayed tiny or absent KV lumen (Figures 3Ee4, F, G). At the 10 somite stage, we used *Tg (sox17:EGFP)* transgenic zebrafish to examine the number of epithelium cells in KV. The results showed that, compared to the control, the number of epithelium cells in KV was significantly reduced in embryos injected with *cdon* MO at the 4-cell stage (Figures 3H, I). Furthermore, we examined cilia development and found that *cdon* morphants displayed slightly shorter cilia than controls (Figures 3Jj2–j4, K), and the number of cilia was also decreased in *cdon* morphants (Figures 3Jj2–j4, L). These results showed that normal expression of *cdon* is required for DFC development and KV morphogenesis, *cdon* MO injection also led to shorter cilia and decreased the number of cilia.

### 3.4 *Cdon* mutation leads to organ LR patterning defect

**FIGURE 4:**
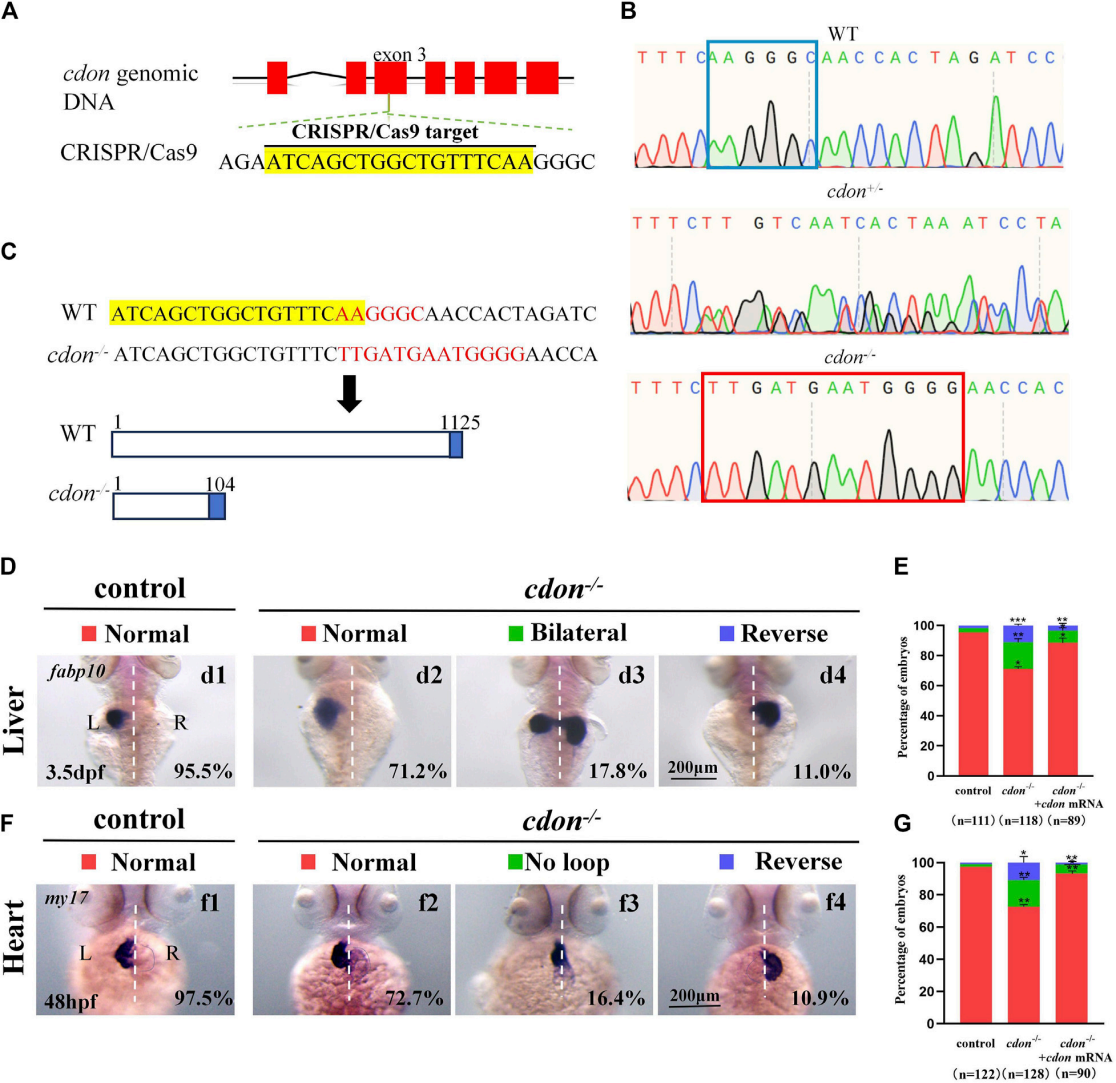
Organ LR patterning defects in *cdon* ^*−/−*^embryos **(A)** CRISPR/Cas9 target was showed. The sequence in the exon 3 of *cdon* gene was chosen as the target (yellow highlighted). **(B)** Sequencing results for the genomic DNA in WT embryos, *cdon* ^*+/−*^ embryos and *cdon* ^*−/−*^ embryos. **(C)** Nucleotide sequences highlighted in yellow are CRISPR/Cas9 targets. In the *cdon* mutant, the sequence “AAGGGC” was changed to “TTGATGAATGGGG”. *Cdon* mutant harbors a frameshift mutation that is predicted to result in the production of truncated Cdon protein (104 amino acids). **(D)** *Cdon* ^*−/−*^ embryos were found to display liver LR defects using WISH. d1, left-sided liver in controls (95.5%, n = 111); d2, left-sided liver in *cdon* ^*−/−*^ embryos (71.2%, n = 118); d3, liver bifida in *cdon* ^*−/−*^ embryos (17.8%, n = 118); d4, right-sided liver in *cdon* ^*−/−*^ embryos (11.0%, n = 118). **(E)** Percentages of left-sided liver, liver bifida, and right-sided liver in *cdon* ^*−/−*^ embryos (n = 118), controls (n = 111) and *cdon* ^*−/−*^ embryos injected with *cdon* mRNA (n = 89), *cdon* ^*−/−*^ embryos show a statistically significant difference compared to controls, and *cdon* ^*−/−*^ embryos injected with *cdon* mRNA show a statistically significant difference compared to *cdon* ^*−/−*^ embryos. **(F)** *Cdon* ^*−/−*^ embryos displayed heart LR defects. f1, normal-loop heart in controls (97.5%, n = 122); f2, normal-loop heart in *cdon* ^*−/−*^ embryos (72.7%, n = 128); f3, no loop heart in *cdon* ^*−/−*^embryos (16.4%, n = 128); f4, reversed loop heart in *cdon* ^*−/−*^ embryos (10.9%, n = 128). The blue dashed line indicates the atrial edge. **(G)** Percentages of normal looping, no looping, and reversed looping of the heart in *cdon* ^*−/−*^ embryos (n = 128), controls (n = 122) and *cdon* ^*−/−*^ embryos injected with *cdon* mRNA (n = 90), *cdon* ^*−/−*^ embryos show a statistically significant difference compared to controls, and *cdon* ^*−/−*^ embryos injected with *cdon* mRNA show a statistically significant difference compared to *cdon* ^*−/−*^ embryos. Statistical analysis was performed using Student’s t-test. “*” *p* < 0.05, “**” *p* < 0.01, “***” *p* < 0.001. Notice: “control” refers to wild-type embryos.

To confirm the role of *cdon* in organ LR patterning, we generated a *cdon* mutant line using the CRISPR-Cas9 method (Shankaran et al., 2017). To generate the *cdon* mutant, we selected a specific sequence in the exon 3 of *cdon* as the target sequence (Figure 4A). As a result, in F1 adults we screened out a frame shift mutation line (Figures 4B, C). In this mutation, the sequence “AAGGGC” in exon 3 of the *cdon* gene was changed to “TTGATGAATGGGG” (Figures 4B, C), resulting in a truncated *Cdon* protein (only 104 amino acids) (Figure 4C). In addition, even though we found that the expression of *cdon* mRNA was greatly downregulated in *cdon* ^−/−^ embryos at the 8-somite stage and 24 hpf (Supplementary Figures S4C, D), the *cdon* ^−/−^ embryos had no distinct external phenotype at different stages (Supplementary Figures S4A, B) and could grow to adulthood. Then we evaluated whether this frameshift mutation leads to liver and heart LR patterning defects using *in situ* experiments. The data showed that *cdon* ^−/−^ embryos, but not control embryos (Figures 4Dd1), displayed liver LR patterning defects: 71.2% of *cdon* ^−/−^ embryos displayed left-sided liver (Figures 4Dd2, E), 17.8% displayed bilateral liver (Figures 4Dd3, E), and 11.0% displayed right-sided liver (Figures 4Dd4, E). Similarly, no heart LR patterning defects were observed in control embryos (Figures 4Ff1, G), but *cdon* ^−/−^ embryos displayed no-loop heart (16.4%; Figures 4Ff3, G) and reversed-loop heart (10.9%; Figures 4Ff4, G). To further confirm the critical role of *cdon* in organ LR patterning, we examined whether injection of *cdon* mRNA could rescue liver and heart LR patterning defects in *cdon* ^*−/−*^ embryos. The data showed that injection of *cdon* mRNA partially restored liver and heart LR patterning (Figures 4E, G). All these data in *cdon* ^−/−^ embryos further demonstrate that *cdon* is essential for organ LR patterning.

### 3.5 KV/cilia-*Nodal/spaw* cascade was also disturbed in *cdon* mutants

**FIGURE 5:**
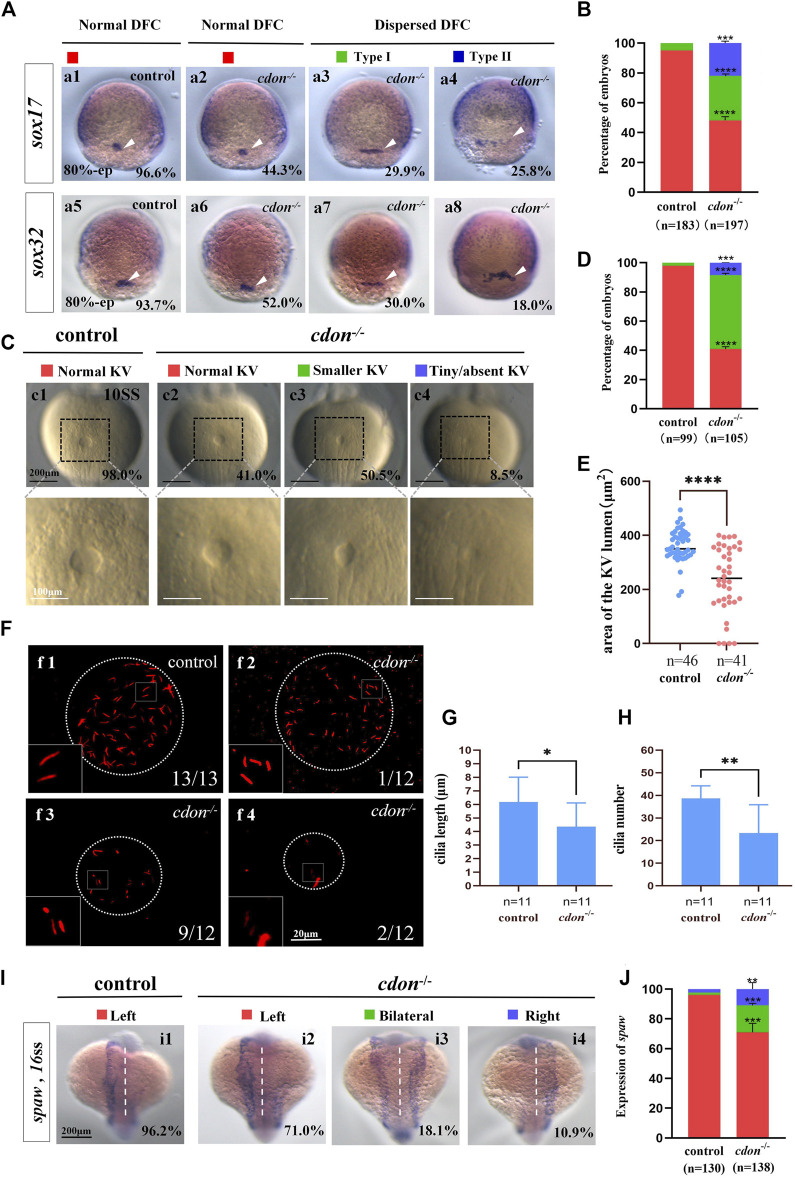
*Cdon* mutation gave rise to defects in KV formation and cilia **(A)** Expression of *sox17* and *sox32* was examined using WISH at 80% epiboly. a1, normal expression of *sox17* in controls (96.6%, n = 88); a2, normal expression of *sox17* in *cdon* ^*−/−*^ embryos (44.3%, n = 97); a3, dispersed expression of *sox17* (type I) in *cdon* ^*−/−*^ embryos (29.9%, n = 97); a4, dispersed expression of *sox17* (type II) in *cdon* ^*−/−*^ embryos (25.8%, n = 97). DFCs (white arrow). a5, normal expression of *sox32* in controls (93.7%, n = 95); a6, normal expression of *sox32* in *cdon* ^*−/−*^ embryos (52.0%, n = 100); a7, dispersed expression of *sox32* (type I) in *cdon* ^*−/−*^ embryos (30%, n = 100); a8, dispersed expression of *sox32* (type II) in *cdon* ^*−/−*^ embryos (18.0%, n = 100). DFCs (white arrow). **(B)** Statistical analysis was performed for DFCs clustering migration in controls (n = 183) and *cdon* ^*−/−*^ embryos (n = 197). Here all the embryos staining with *sox17* or *sox32* were used together to calculate the percentage. Normal DFC and dispersed DFC show significant differences between control embryos and *cdon* ^*−/−*^ embryos **(C,D)** Morphology of KV in different group of embryos at 10sss. c1, normal KV in controls (98.0%, n = 99); c2, normal KV in *cdon* ^*−/−*^ embryos (41.0%, n = 105, *p* < 0.0001); c3, smaller KV in *cdon* ^*−/−*^ embryos (50.5%, n = 105, *p* < 0.0001); c4, tiny/absent KV in *cdon* ^*−/−*^ embryos (8.5%, n = 105, *p* < 0.001). “Normal KV” represents a KV lumen area greater than 300 μm^2^, “smaller KV” represents a KV lumen area between 100 and 300 μm^2^, and “tiny/absent KV” represents a KV lumen area less than 100 μm^2^. **(E)** Area of the KV lumen (µm^2^) in control and *cdon* ^*−/−*^ embryos. “n” represents the sample size. **(F)** The number and the length of cilia were examined at 10 sss. f1, cilia in controls; f2-f4, cilia in *cdon* ^*−/−*^ embryos **(G)** Statistical chart for cilia length in KV. “n” represents the sample size. **(H)** Statistical chart for cilia number in KV. “n” represents the sample size. **(I,J)** Expression of *spaw* in controls and *cdon* ^*−/−*^ embryos. i1, left-sided *spaw* in controls (96.2%, n = 130); i2, left-sided *spaw* in *cdon* ^*−/−*^ embryos (71.0%, n = 138, *p* < 0.001); i3, bilateral *spaw* in *cdon* ^*−/−*^ embryos (18.1%, n = 138, *p* < 0.001); i4,right-sided *spaw* in *cdon* ^*−/−*^ embryos (10.9%, n = 138, *p* < 0.01). Statistical analysis was performed using Student’s t-test. “*” *p* < 0.05, “**” *p* < 0.01, “***” *p* < 0.001, “****” *p* < 0.0001. Notice: “control” refers to wild-type embryos.

To further confirm the mechanism by which *cdon* regulates organ LR patterning, we also examined whether the KV/cilia-*Nodal/spaw* cascade was affected in *cdon* ^−/−^ embryos. First, we examined the expression of *sox17* and *sox32* at the 80% epiboly stage to evaluate whether the clustering DFC migration is disturbed in *cdon* ^*−/−*^ embryos. The data showed that 55.7% of *cdon* ^*-/-*^embryos displayed dispersed expression of *sox17* (Figures 5Aa3, a4, B), and 48.0% of *cdon* ^*−/−*^ embryos displayed dispersed expression of s*ox32* (Figures 5Aa7, a8, B). In contrast, the expression of *sox17* and *sox32* was normal in control embryos (Figures 5Aa1, Aa5, B). This data indicated that clustering DFC was disturbed in *cdon* ^*−/−*^ embryos. Then we evaluated whether KV morphogenesis and cilia development were affected in *cdon* ^*−/−*^ embryos. The data showed that in many *cdon* ^−/−^ embryos, the size of the KV lumen became smaller or absent (Figures 5Cc3, c4, D, E). Similar to *cdon* morphants, in *cdon* ^−/−^ embryos, the cilia length is slightly shorter (Figures 5Ff1–f4, G) and the cilia number is decreased (Figures 5Ff1–f4, H). Finally, we examined the expression pattern of *spaw* in control and *cdon* ^*−/−*^ embryos. The data showed that left-sided *spaw* expression was also disturbed in *cdon* ^−/−^ embryos, displaying left-sided *spaw* (Figures 5Ii2, J), bilateral *spaw* (Figures 5Ii3, J), and right-sided *spaw* (Figures 5Ii4, J). In addition, the expression of the *Nodal/spaw* downstream genes *lefty1* and *lefty2* was also disturbed in *cdon* ^*−/−*^ embryos (Supplementary Figures 5A–D). These data further confirm that the KV/cilia-*Nodal/spaw* cascade may mediate the regulation of heart and liver LR patterning by *cdon* during early development.

### 3.6 *Cdon* loss of function in DFCs results in organ LR patterning defects

**FIGURE 6:**
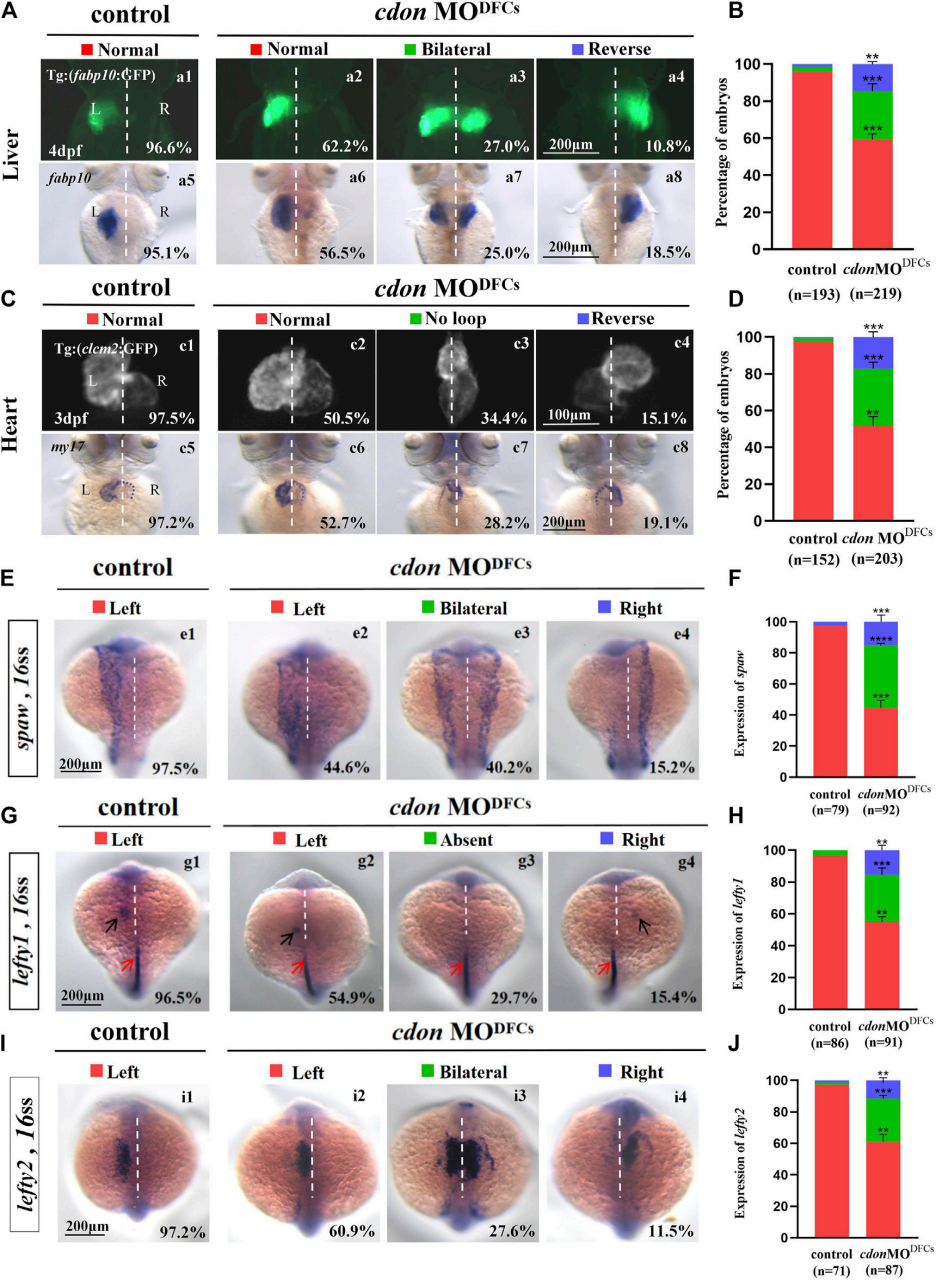
Organ left-right patterning defects in embryos injected with *cdon* MO at the 256-cell stage **(A)** Embryos injected with *cdon* MO at the 256-cell stage were found to cause liver LR defects. a1, normal liver in *Tg(fabp10:GFP)* transgenic controls (96.6%, n = 90); a2, normal liver in *Tg(fabp10:GFP)* transgenic embryos injected with *cdon* MO at the 256-cell stage (62.2%, n = 111); a3, liver bifida in *Tg(fabp10:GFP)* transgenic embryos injected with *cdon* MO at the 256-cell stage (27.0%, n = 111); a4, reversed liver in *Tg(fabp10:GFP)* transgenic embryos injected with *cdon* MO at the 256-cell stage (10.8%, n = 111). a5, normal liver in wild-type controls (95.1%, n = 103); a6-a8, wild-type embryos injected with *cdon* MO at the 256-cell stage were examined for liver laterality at 4 dpf by WISH against *fabp10* probe (n = 108). **(B)** Percentages of normal liver, liver bifida, and reversed liver in control embryos and embryos injected with *cdon* MO at the 256-cell stage. Embryos injected with *cdon* MO at the 256-cell stage show a statistically significant difference compared to controls. **(C)** Heart morphogenesis in *Tg(cmcl2:GFP)* transgenic embryos and wild-type embryos injected with *cdon* MO at the 256-cell stage. c1, normal-loop in *Tg(cmcl2:GFP)* transgenic controls (97.5%, n = 80); c2, normal-loop in *Tg(cmcl2:GFP)* transgenic embryos injected with *cdon* MO at the 256-cell stage (50.5%, n = 93); c3, no loop in *Tg(cmcl2:GFP)* transgenic embryos injected with *cdon* MO at the 256-cell stage (34.4%, n = 93); c4, reversed-loop in *Tg(cmcl2:GFP)* transgenic embryos injected with *cdon* MO at the 256-cell stage (15.1%, n = 93); c5, normal-loop in wild type controls (97.2%, n = 72); c6-c8, wild-type embryos injected with *cdon* MO at 256-cell stage were examined for cardiac looping at 72 hpf by WISH against *my17* (n = 110). The blue dashed line indicates the atrial edge. **(D)** Percentages of normal looping, no looping, and reversed looping of the heart in all the embryos injected with or without *cdon* MO at the 256-cell stage. Embryos injected with *cdon* MO at the 256-cell stage show a statistically significant difference compared to controls. **(E,F)** Expression of *Nodal/spaw* in controls (n = 79) and embryos injected with *cdon* MO at the 256-cell stage (n = 92). **(G,H)** Expression of *lefty1* in controls (n = 86) and embryos injected with *cdon* MO at the 256-cell stage (n = 91). **(I,J)** Expression of *lefty2* in controls (n = 71) and embryos injected with *cdon* MO at the 256-cell stage (n = 87). Statistical analysis was performed using Student’s t-test. “**” *p* < 0.01, “***” *p* < 0.001, “****” *p* < 0.0001. Notice: in “B” and “D”, all the transgenic embryos and wild-type embryos were used together to calculate the percentage. “Control” refers to wild-type embryos that were not injected with *cdon* MO.

Our data have shown that *cdon* is expressed not only in DFCs and KV epithelial cells but also in other cells such as those in the midline and PSM (Figure 1A; Supplementary Figures S4C, D). To confirm whether *cdon* specifically regulates organ LR patterning via the DFCs-KV/cilia-*Nodal/spaw* cascade, we injected *cdon* MO at the 256–512 cell stage to predominantly block the translation of *cdon* mRNA in DFCs (Amack and Yost, 2004; Zhu et al., 2019), then evaluated whether organ LR patterning was disturbed. Indeed, in the embryos injected with *cdon* MO at the 256–512 cell stage, liver and heart LR patterning were disturbed (Figures 6A–D). In liver transgenic embryos *Tg* (*fabp10*:*GFP*), heart transgenic embryos *T*g (*cmlc2*:*GFP*) and wild-type embryos, after predominantly down-regulating the function of *cdon* in DFCs, the liver (Figures 6A, B) and heart (Figures 6C, D) LR patterning are all disturbed. Next, we evaluated whether the DFCs-KV/cilia cascade was affected after predominantly down-regulating the function of *cdon* in DFCs. The data showed that clustering DFC was disturbed (Supplementary Figures S6Aa1-a8, B), the size of the KV lumen was smaller (Supplementary Figures S6Cc1-c4, D-E), the length of cilia was slightly shorter (Supplementary Figures S6Ff1-f4,G), and the number of cilia was also decreased (Supplementary Figures S6Ff1-f4, H). Finally, we evaluated whether Nodal/spaw signaling was disturbed in embryos injected with *cdon* MO at the 256–512 cell stage. As a result, the expression of left-sided *Nodal/spaw* and its downstream genes *lefty1* and *lefty2* was also randomized in embryos injected with *cdon* MO at the 256–512 cell stage (Figures 6E–J). Regarding the expression of *spaw*, 44.6% (Figures 6Ee2), 40.2% (Figures 6Ee3), and 15.2% (Figures 6Ee4) of embryos injected with *cdon* MO displayed left-sided *spaw*, both-sided *spaw*, and right-sided *spaw*, respectively, while 97.5% of control embryos displayed left-sided *spaw* (Figures 6Ee1). Regarding the expression of *lefty1*, 54.9% (Figures 6Gg2), 29.7% (Figures 6Gg3), and 15.4% (Figures 6Gg4) of embryos injected with *cdon* MO displayed left-sided *lefty1*, absent *lefty1*, and right-sided *lefty1*, respectively, while 96.5% of control embryos displayed left-sided *lefty1*. Regarding the expression of *lefty2*, 60.9% (Figures 6Ii2), 27.6% (Figures 6Ii3), and 11.5% (Figures 6Ii4) of embryos injected with *cdon* MO displayed left-sided *lefty2*, both-sided *lefty2*, and right-sided *lefty2*, respectively, while 97.2% of control embryos displayed left-sided *lefty2* (Figures 6Ii1). These data indicate that left-sided Nodal/spaw signaling was randomized after down-regulating the function of *cdon* in DFCs. In conclusion, all these data suggest that *cdon* specifically regulates organ LR patterning via the DFCs-KV/cilia-*Nodal/spaw* cascade.

## 4 Discussion

Mouse node/cilia [referred to as the “left-right organizer” (LRO)] was first identified to play a critical role during organ LR patterning (Sulik et al., 1994; Nonaka et al., 1998; Schneider et al., 1999; Kajikawa et al., 2022). Similar to mice, transient structures were identified in other vertebrate embryos such as zebrafish, suggesting a conserved cilia-based mechanism regulating LR patterning in zebrafish (Essner et al., 2002). Functional studies confirmed the presence of motile cilia and asymmetric fluid flow in Kupffer’s vesicle in zebrafish (Essner et al., 2005), and genetic or embryological perturbation of these ciliated structures disrupted asymmetric Nodal pathway expression and organ laterality (Essner et al., 2005; Amack, 2014; Kuhns et al., 2019; Liu et al., 2019). In zebrafish, the transgenic line *Tg (sox17:EGFP)* was developed to label the DFC/KV cells (Chung and Stainier, 2008), and several developmental steps have been identified to build a functional KV/Cilia. DFCs appear at mid-epiboly stage, migrate, proliferate, and then undergo a mesenchymal-to-epithelial transition to form the KV in early somite stage (Forrest et al., 2022). The KV develops directional fluid flow and establishes LR signaling, and then breaks down around 18 hpf when KV cells undergo an epithelial-to-mesenchymal transition (Amack, 2021) and migrate to incorporate into muscle and notochord (Ikeda et al., 2022). In the past decades, many genes and environmental elements have been reported to be involved in DFC clustering, migration (Ablooglu et al., 2010; Gao et al., 2011; Lai et al., 2012; Kajikawa et al., 2022; Liu et al., 2022), proliferation (Zhang et al., 2012; Gokey et al., 2015; Liu et al., 2019; Abdel-Razek et al., 2023), and the final KV formation and ciliogenesis. However, the mechanisms underlying this process are far from being completely elucidated.

*Cdon* is a cell surface glycoprotein that belong to a subgroup of the immunoglobulin (Ig) superfamily of cell adhesion molecules (Sanchez-Arrones et al., 2012). The role of *cdon* in organ development and function has been reported in many literature, including its role in neural differentiation, migration and survival (Jeong et al., 2014; Powell et al., 2015; Wang et al., 2017; Uluca et al., 2022; Kim et al., 2023), cardiac remodeling and fibrosis (Jeong et al., 2017) and myoblast fusion (Castiglioni et al., 2018). More recently, in zebrafish, *cdon* was reported to be involved in trunk neural crest cell migration, slow-twitch muscle development (Lencer et al., 2023), and limb growth (Echevarria-Andino et al., 2023). However, whether it plays a critical role in organ LR patterning at earlier stage has not been reported.

Here, we identified that *cdon* is expressed in DFCs during the gastrulation movement (Figures 1Aa2–a5) and in the epithelial cells of the KV at the early somitogenesis stage (Figures 1Aa6, a7). Further data suggested that *cdon* loss of function leads to defect in DFC clustering and decreases DFCs number. These defects are correlated with disturbances in KV formation, cilia number and the subsequent organ LR patterning. Therefore, during embryonic development, in addition to its role in regulating trunk neural crest cell migration (Lencer et al., 2023) and defining the correct proximo-distal patterning of eye development (Cardozo et al., 2014), we identified an earlier role of *cdon* in regulating LR patterning. Comparing our data in *cdon* morphants and *cdon* mutants, we discovered that the organ LR patterning defect in *cdon* morphants is stronger than that in *cdon* mutants. Given the well-known genetic compensation response in zebrafish (Ma et al., 2019), the possible reason is the mutation of *cdon* may upregulate other genes to compensate for the loss of *cdon* function. This possibility needs further work to elucidate.

Even our work identified the role of *cdon* in organ LR patterning, the detailed mechanism underlying was not elucidated. In our data, we found that the number of DFCs and epithelium cells in KV was decreased (Figures 3C, D, H, I), as well the cohesive migration was defect in *cdon* mutants (Figures 3A–D), so the genes being relative to cell migration, cell proliferation/differentiation or cell apoptosis should be affected in *cdon* mutants. In zebrafish, reduction of Wnt signaling leads to a disruption of LR patterning, shorter and fewer cilia (Caron et al., 2012), depletion of β-catenin 1 or β-catenin 2 in DFCs/KV leads to poor KV cell proliferation, abnormal cilia formation (Zhang et al., 2012). On the contrary, knockdown of Autotaxin/Lpar3 signaling activates β-catenin and also compromises DFC cohesive migration, KV formation and ciliogenesis (Lai et al., 2012). These reports suggested that both downregulation and upregulation of Wnt signaling would give rise to defect in KV morphogenesis, abnormal cilia formation and the sequential organ LR patterning. Importantly, early reports in mouse demonstrated that Cdon negatively regulates Wnt signaling during neural development and cardiac remodeling (Jeong et al., 2014; Jeong et al., 2017), the Cdo-deficient dorsal forebrain displays stronger Wnt signalling activity, increased cell proliferation and enhanced expression of the dorsal markers and Wnt targets (Jeong et al., 2014). These literature suggested the possibility that in zebrafish, loss of *cdon* function upregulates Wnt signaling, which compromises DFC cohesive migration, KV formation, ciliogenesis and the sequential organ LR patterning. While far more work is needed to clarify whether Wnt signaling lies downstream of *cdon* to regulate LR patterning.

## Data Availability

The original contributions presented in the study are included in the article/Supplementary Material, further inquiries can be directed to the corresponding authors.
