# Supplementary material for: Cdon is essential for organ left-right patterning by regulating dorsal forerunner cells clustering and Kupffer’s vesicle morphogenesis
Source: Front Cell Dev Biol. 2024 Aug 22;12:1429782. doi: 10.3389/fcell.2024.1429782 (PMC11374761; doi:10.3389/fcell.2024.1429782)
Supplement: Supplementary file 1 [file DataSheet1.pdf]

Figure S1-S6 and Legends

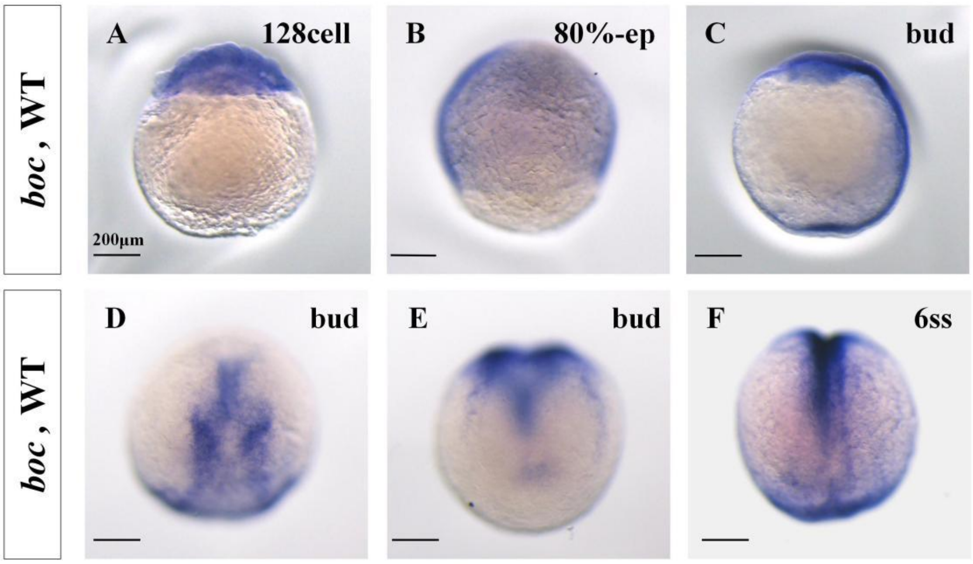

Figure S1. The expression of *boc* at different developmental stages

(A) *boc* was equally distributed in all the cells at 128 cell stage. (B) The expression of *boc* was examined at 80% epiboly stage. (C-E) The expression of *boc* at bud stage. Lateral view (C); Head in dorsal view, *boc* was enriched in presumptive neural crest but not in midline (D); Tail, dorsal view. (E) *boc* was examined at 6ss, it was not expressed in KVs.

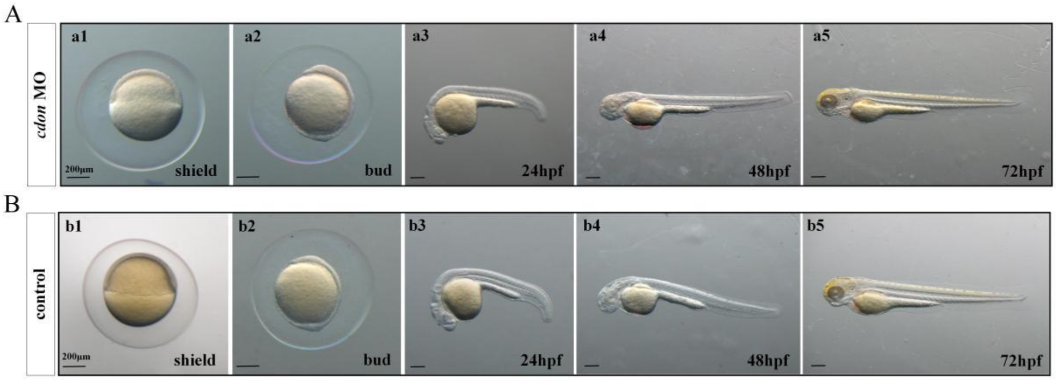

Figure S2. The external phenotype in embryos injected with *cdon* MO

(A) Lateral view of embryos injected with *cdon* MO at shield stage (a1), bud stage (a2), 24 hpf (a3), 48 hpf (a4) and 72 hpf (a5). **(B)** Lateral view of control embryos at shield stage (b1), bud stage (b2), 24 hpf (b3), 48 hpf (b4) and 72 hpf (b5). There is no distinct difference between controls and embryos injected with *cdon* MO. Notice: "control" refers to wild-type embryos that were not injected with *cdon* MO.

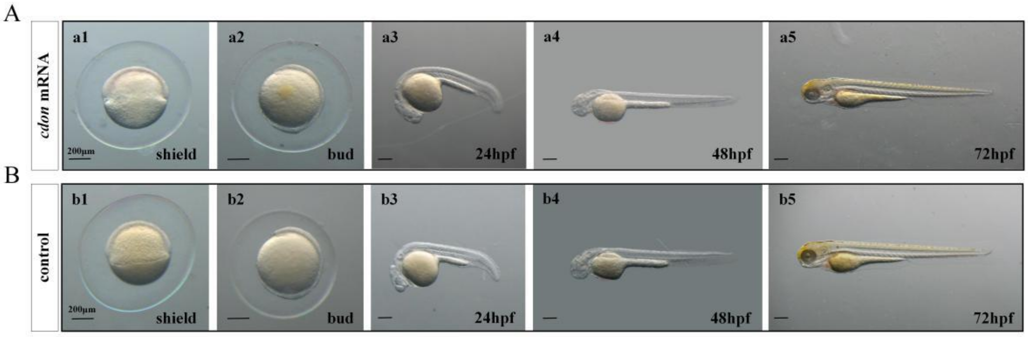

Figure S3. The external phenotype in embryos injected with *cdon* mRNA

(A) Lateral view of embryos injected with *cdon* mRNA (15ng/μl) at shield stage (a1), bud stage (a2), 24 hpf (a3), 48 hpf (a4) and 72 hpf (a5). **(B)** Lateral view of control embryos at shield stage (b1), bud stage (b2), 24 hpf (b3), 48 hpf (b4) and 72 hpf (b5). There is no distinct difference between controls and embryos injected with *cdon* mRNA (15ng/μl). Notice: “control” refers to wild-type embryos that were not injected with *cdon* mRNA.

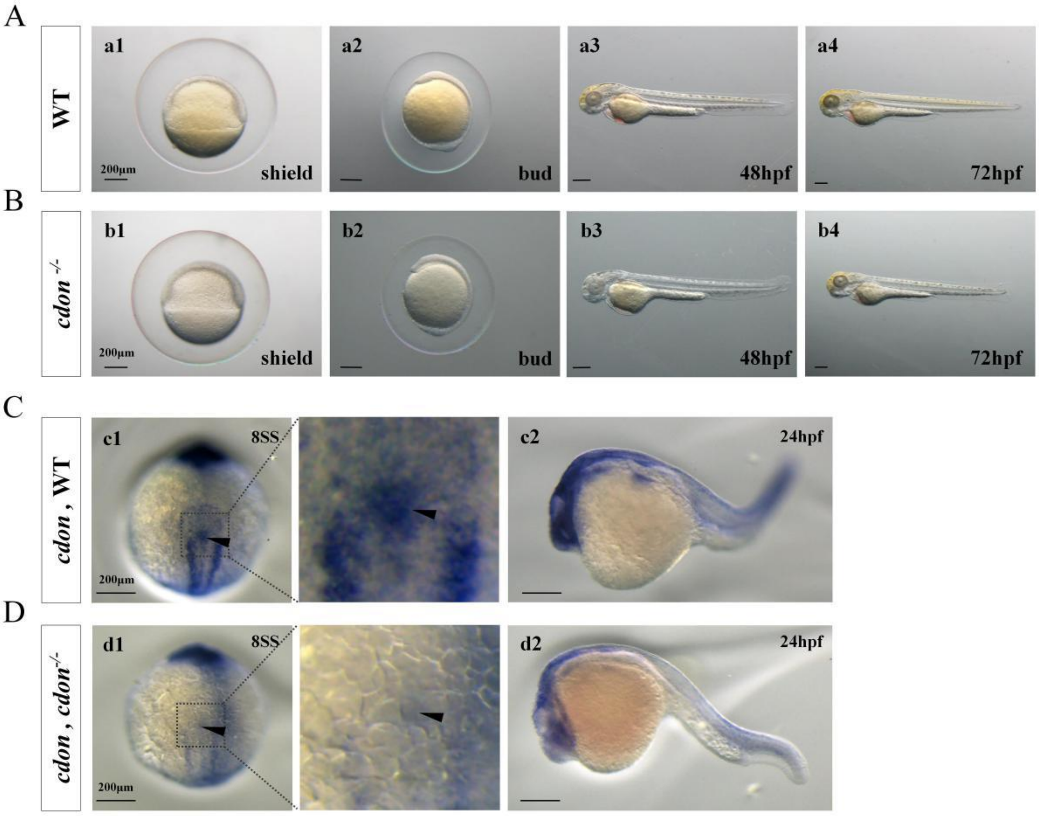

Figure S4. The external phenotype and the expression of *cdon* in controls and *cdon*$^{-/-}$embryos

(A) The lateral view of wild type embryos at shield stage (a1), bud stage (a2), 48 hpf (a3) and 72 hpf (a4). **(B)** The lateral view of *cdon*$^{-/-}$ embryos at shield stage (b1), bud stage (b2), 48 hpf (b3) and 72 hpf (b4). No distinct difference was found between wild type embryos and *cdon*$^{-/-}$ embryos at each stage (A, B). **(C)** The expression of *cdon* was examined at 8 somite stage (c1) and 24 hpf (c2). *Cdon* was expressed in the epithelial cells in KV (c1, arrow head showing). **(D)** The expression of *cdon* in the epithelial cells in KV was decreased (d1, arrow head showing), and it's expression was also decreased at 24 hpf (d2).

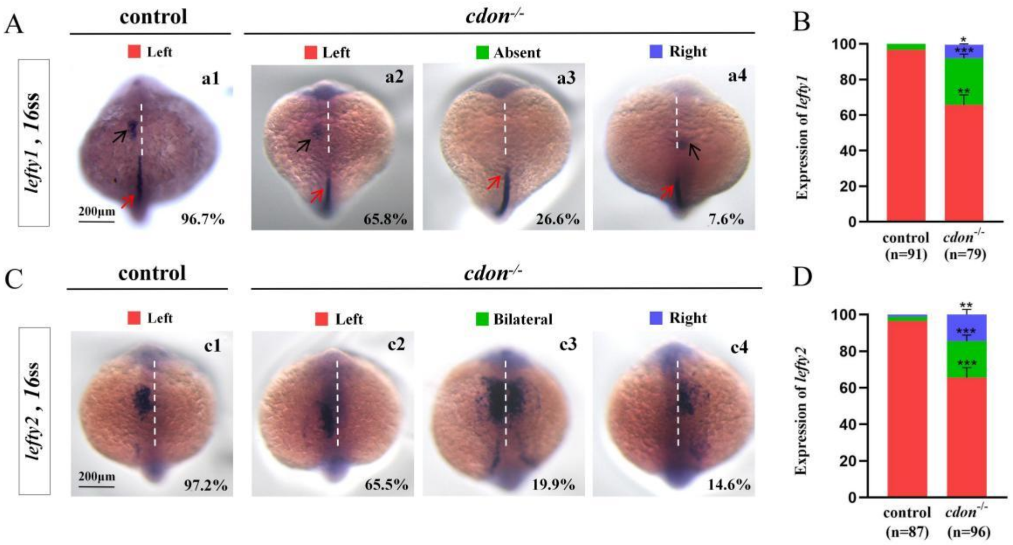

Figure S5. The expression of *lefty1* and *lefty2* in controls and *cdon*$^{-/-}$ embryos

(A, B) The expression of *lefty1* in wild type embryos (a1, n=91). Left-sided *lefty1* (a2, n=79, $p < 0.01$), disappeared *lefty1* (a3, n=79, $p < 0.001$) and right-sided *lefty1* (a4, n=79, $p < 0.05$) were observed in heart filed of *cdon*$^{-/-}$ embryos . Black arrow showed the heart field, red arrow showed the middle line. **(C,D)** In most of wild type embryos, *lefty2* was observed in the left side of the embryos (c1, n=87). Left-sided *lefty2* (c2, n=96, $p < 0.001$), bilateral expression of *lefty2* (c3, n=96, $p < 0.001$) and right-sided *lefty2* (c4, n=96, $p < 0.01$) were observed in *cdon*$^{-/-}$ embryos. Statistical analysis was performed using Student's t-test. "*" $p < 0.05$, "**" $p < 0.01$, "***" $p < 0.001$. Notice: "control" in "B", "D" refers to wild-type zebrafish embryos.

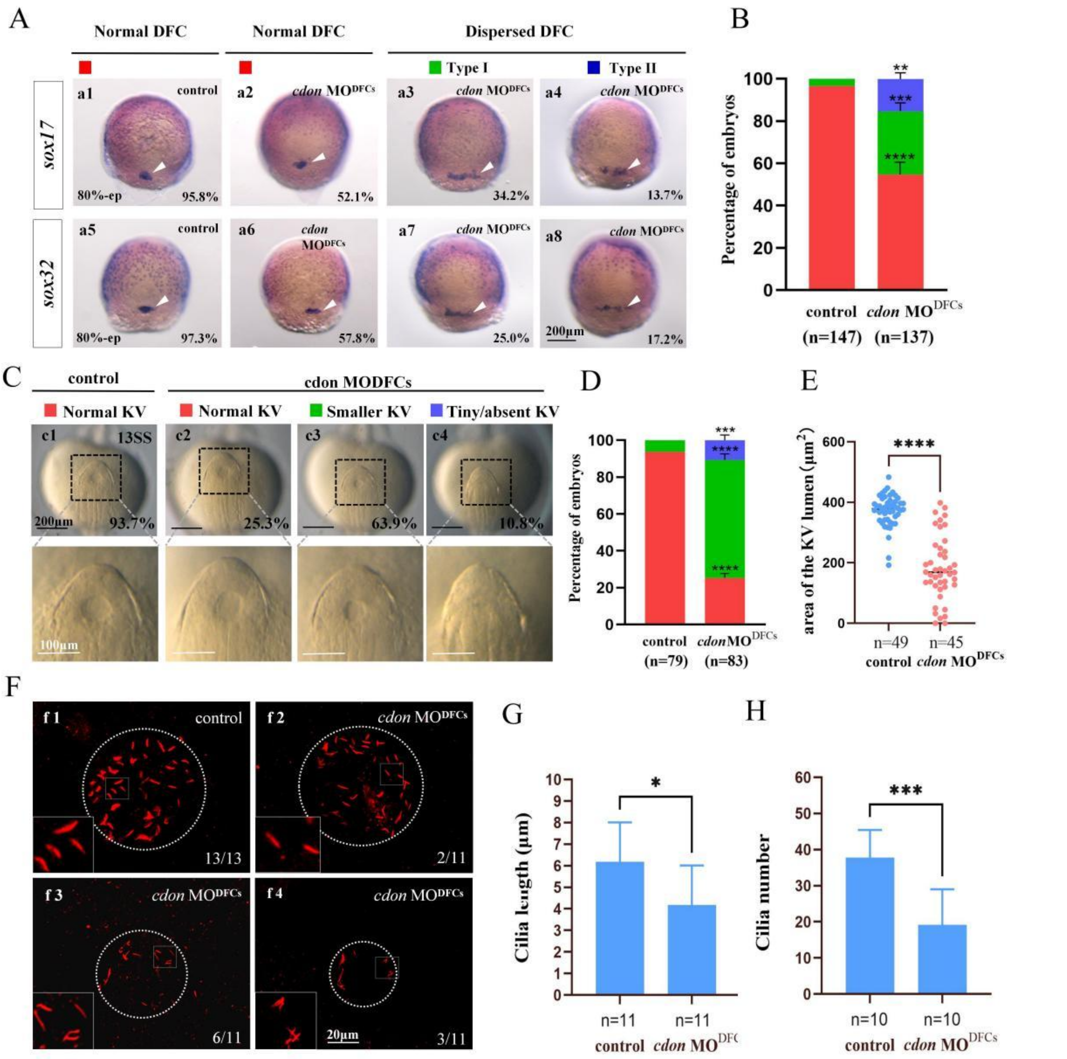

Figure S6. Injection of *cdon* MO at the 256-cell stage disturbed clustering DFCs, KV morphogenesis, cilia number and cilia length

(A) Expression of *sox17* and *sox32* was examined using WISH at 80% epiboly. a1, normal expression of *sox17* in control embryos (95.8%, n=72); a2, normal expression of *sox17* in embryos injected with *cdon* MO at the 256-cell stage (52.1%, n=73); a3, dispersed expression of *sox17* (type I) in embryos injected with *cdon* MO at the 256-cell stage (34.2%, n=73); a4, dispersed expression of *sox17* (type II) in embryos injected with *cdon* MO at the 256-cell stage (13.7%, n=73). DFCs (white arrow). a5, normal expression of *sox32* in control embryos (97.3%, n=75); a6, normal expression of *sox32* in embryos injected with *cdon* MO at the 256-cell stage (57.8%, n=64); a7, dispersed expression of *sox32* (type I)in embryos injected with *cdon* MO at the 256-cell stage (25.0%, n=64); a8, dispersed expression of *sox32* (type II)in embryos injected with *cdon* MO at the 256-cell stage 17.2%, n=64). DFCs (white arrow). **(B)** Percentages of normal *sox17*, dispersed *sox17*, normal *sox32* and dispersed *sox32* in embryos injected with or without *cdon* MO at the 256-cell stage. Here all the embryos staining with *sox17* or *sox32* were used together to

calculate the percentage. Normal DFC and dispersed DFC show significant differences between control embryos and embryos injected with *cdon* MO at the 256-cell stage **(C-D)** Morphology of KV at 13ss. c1,normal KV in control embryos (93.7%, n=79); c2, normal KV in embryos injected with *cdon* MO at the 256-cell stage (25.3%, n=83, $p < 0.0001$); c3, smaller KV in embryos injected with *cdon* MO at the 256-cell stage (63.9%, n=83, $p < 0.0001$); c4, tiny/absent KV in embryos injected with *cdon* MO at the 256-cell stage (10.8%, n = 83, $p < 0.001$). "Normal KV" represents a KV lumen greater than 300 μm^2, "smaller KV" represents a KV lumen area between 100-300 μm^2, and "tiny/absent KV" represents a KV lumen area less than 100 μm^2. **(E)** Area of the KV lumen (μm^2) in control and embryos injected with *cdon* MO at the 256-cell stage. "n" represents the sample size. **(F)** Number and length of cilia at 10ss. e1, cilia in control embryos; f2-f4, cilia in embryos injected with *cdon* MO at the 256-cell stage. **(G)** Statistical chart for cilia length in KV. "n"represents the sample size. **(H)** Statistical chart for cilia number in KV. "n" represents the sample size. Statistical analysis was performed using Student's t-test. "*" $p < 0.05$, "**" $p < 0.01$, "***" $p < 0.001$, "****" $p < 0.0001$.
